# STAR (stroma-tumor AI risk) assessment: association of AI-derived tumor-stroma proportion with patient survival provides added prognostic value beyond KELIM in epithelial ovarian cancer

**DOI:** 10.1038/s44276-026-00205-1

**Published:** 2026-02-06

**Authors:** Arpit Aggarwal, Morgann Madill, Mayukhmala Jana, Tilak Pathak, Timothy K. Starr, Boris Winterhoff, Katelyn M. Tessier, Britt K. Erickson, Andrew C. Nelson, Emil Lou, Anant Madabhushi, Martina Bazzaro

**Affiliations:** 1 Department of Biomedical Engineering, Emory University and Georgia Institute of Technology, Atlanta, GA USA; 2 Masonic Cancer Center and Department of Obstetrics, Gynecology and Women’s Health, University of Minnesota, Minneapolis, MN USA; 3 Masonic Cancer Center, Biostatistics Core, Minneapolis, MN USA; 4 Department of Laboratory Medicine & Pathology, University of Minnesota, Minneapolis, MN USA; 5 Division of Hematology, Oncology, and Transplantation, Department of Medicine, University of Minnesota, Minneapolis, MN USA; 6 Atlanta Veterans Administration Medical Center, Atlanta, GA USA; 7 Department of Biomedical and Clinical Sciences (BKV), Linköping University, Linköping, Sweden

## Abstract

**Background:**

There remains a critical need for prognostic biomarkers of treatment response in epithelial ovarian cancer (EOC). The KELIM score, derived from the rate of CA-125 elimination during the first 100 days of treatment, is a clinically available biomarker of treatment response to platinum-based chemotherapy, its utility is limited by the need for post-treatment data. Tumor–stroma proportion (TSP) has emerged as a prognostic biomarker across several malignancies. Studies from our group have shown that high TSP (≥50% stroma content assessed by pathologist evaluation, TSP_manual_) is associated with platinum resistance and poor survival in EOC at diagnosis and before treatment.

**Methods:**

We compared the prognostic value of TSP and KELIM by analyzing manual pathologist (TSP_manual_) and artificial intelligence–derived assessments (TSP_auto_) on digitized images from a cohort of EOC specimens.

**Results:**

In this cohort, we showed the prognostic significance of TSP_manual_, confirming prior findings. Furthermore, TSP_auto_ and TSP_manual_ assessments were highly concordant (94% agreement, Cohen’s Kappa 0.89, *p*<0.001), providing a highly reproducible, automated approach. Unlike KELIM, which was only associated with platinum resistance, high TSP_auto_ was significantly associated with poor survival (HR 1.99, *p* = 0.02).

**Conclusion:**

These findings support AI-derived TSP as a pre-treatment prognostic biomarker for EOC that complements KELIM.

## Introduction

Ovarian cancer remains one of the deadliest gynecologic malignancies, with high recurrence rates and over a third of patients developing resistance to the standard of treatment—platinum-based chemotherapy [1, 2]. Biomarkers that predict therapeutic response are essential for optimizing treatment strategies and improving patient outcomes [1]. One increasingly utilized tool in epithelial ovarian cancer (EOC) is the KELIM score (constant elimination rate of CA-125) [3, 4]. KELIM is calculated using serum levels of the tumor marker CA-125 during initial platinum-based chemotherapy [3, 4]. To date, the KELIM score has demonstrated independent predictive value for response to initial platinum-based chemotherapy, maintenance therapies [3, 4], and survival outcomes [4–7]. KELIM scoring requires at least three CA-125 measurements within the first 100 days of therapy, which can be entered into a free online calculator [8]. A KELIM score of greater than or equal to 1 is considered favorable and is associated with longer progression-free survival (PFS), overall survival (OS), and platinum sensitivity [9]. Conversely, a KELIM score of less than 1 is linked to poorer survival outcomes and platinum resistance [9]. KELIM offers advantages over other biomarkers in the field due to its cost-effectiveness, accessibility, and ease of use for clinicians in predicting response to initial chemotherapy [9].

Tumor stroma proportion (TSP) is an emerging histopathological marker for prognostication and treatment planning in various solid tumors [10–15], including ovarian cancer [15–17]. Our group has retrospectively and prospectively demonstrated its utility as a predictor of chemoresistance in EOC [18, 19]. TSP is defined as the proportion of stromal to cancerous tissue within a tumor and serves as a potential prognostic parameter [17–20]. A low TSP, indicating a smaller proportion of stromal tissue relative to tumor cells, is associated with a more favorable prognosis [18, 19]. Conversely, a high TSP—reflecting a greater stromal component—typically correlates with a more aggressive tumor phenotype and poorer prognosis [18, 19]. A recent publication reaffirmed the prognostic value of TSP in a clinical trial ovarian cancer population treated with chemotherapy and immunotherapy, and associated stroma-rich TSP tumors with immunosuppressive microenvironment [21]. With continued studies such as this, accurate TSP assessment through histopathological examination may help guide therapeutic decisions, such as choosing between targeted therapies and conventional treatments for EOC management.

Unlike KELIM, which requires post-treatment CA-125 measurements [1, 22], tumor–stroma proportion (TSP) is a simple, low-cost biomarker that can be assessed on routine surgical specimens obtained at initial diagnosis by biopsy or upfront surgery. To improve reproducibility and reduce the burden of manual scoring, computational pathology methods using artificial intelligence (AI) have been investigated to quantify TSP in several solid tumors, including gastric, colorectal, and pancreatic cancers [23–27] However, AI-based TSP assessment has not yet been evaluated in EOC, where a reliable pre-treatment prognostic biomarker is critically needed. This study aimed to determine whether AI-derived TSP, measurable at diagnosis, could complement or surpass KELIM in its association with survival outcomes in EOC.

## Methods

### Dataset description

**Fig. 1:**
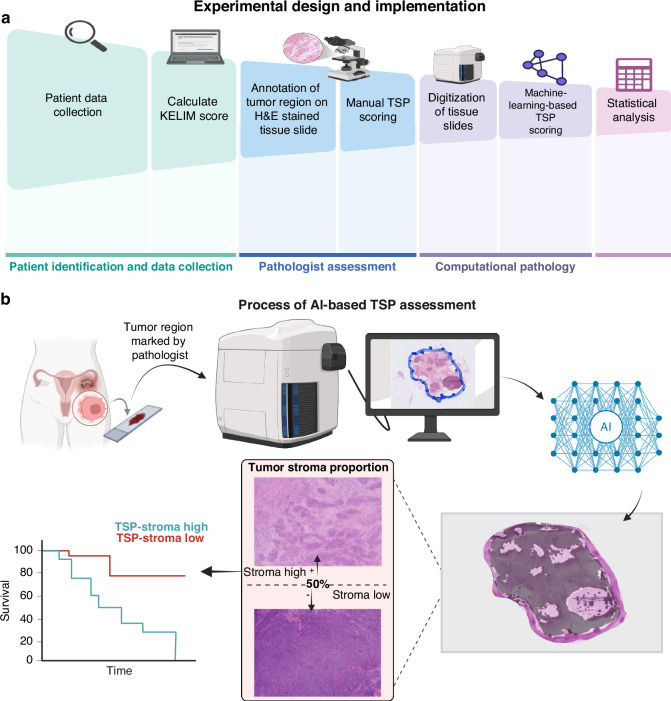
Overall workflow. **a** Experimental design flowchart. **b** General procedures of TSP_auto_ assessment from specimen collection, conversion to digital pathology images, and TSP assessment by pretrained model. Qualitative examples of AI-based segmentation and examples of high and low TSP tissue samples are shown. Created in https://BioRender.com.

The curated cohort included 89 patients diagnosed with EOC at the University of Minnesota between April 2014 and October 2024. All patients had available tissue slides for analysis, at least three CA-125 measurements taken within 100 days of starting a platinum-based chemotherapy regimen, and survival outcome data. Survival analysis was considered at two points: overall survival (OS) and progression-free survival (PFS). OS was defined as the time from date of surgery to the date of death or were censored at last contact. PFS was defined as the time from the date of primary surgery or biopsy to radiologic or biopsy proven recurrence and were censored at date of last contact. Platinum-resistant status was determined based on standard criteria, defined as disease progression during or within 180 days of completing platinum-based chemotherapy [28]. The processes from patient data collection to manual and AI-based TSP assessment and statistical analysis is summarized in Fig. 1.

### Calculation of the KELIM score

The validated online tool developed by You et al. [8], which calculates the kinetic elimination rate of CA-125 and is freely available, was used to determine KELIM for patients receiving adjuvant chemotherapy and NACT [9]. To use the KELIM tool, users must input the date of each chemotherapy cycle and the CA-125 levels measured within 100 days of initiating platinum-based chemotherapy. The algorithm then generates a KELIM score, which was recorded during data collection.

### Manual tumor-stroma-proportion assessment (TSP_manual_)

All digitized hematoxylin and eosin (H&E)–stained slides were reviewed by a board-certified anatomic pathologist for quality control and to delineate tumor-containing regions. Pathologist were blinded to any patient characteristics or clinical findings including original histologic diagnosis, KELIM score, stage, or outcomes. Areas of necrosis and normal tissue were excluded, and entire tumor regions were marked to guide subsequent digital algorithm analysis. When multiple slides with representative tumor were available for a given case, preference was given to slides from the primary tumor site. If primary site material was unavailable, metastatic lesions from the omentum or peritoneum were selected instead, as these sites are commonly sampled and used for diagnosis in routine clinical practice. Tumor regions were identified by visual inspection at 10x magnification, the tumor region of interest (for both TSP_manual_ and TSP_auto_) was marked by the pathologist, then slides were scanned and digitized at 40x resolution for AI-based analysis.

Manual TSP scores (TSP_manual)_ were obtained following the same procedure described by Lou et al [19]. Pathologists reviewed the H&E slides at 10x magnification, identified tumor areas, assessed the relative proportions of stromal tissue and cancer cells, and categorized TSP_manual_ as high (≥50%) or low (<50%) using the previously validated 50% cutoff [10–12, 14, 17, 18].

### Artificial intelligence-based tumor-stroma-proportion assessment

A pretrained deep learning model using a U-Net architecture segmented the tumor and stromal areas within these marked regions, comprising 14,788,929 parameters [29]. This model, trained and validated on 212 well-annotated oral cavity tissue microarray images, demonstrated consistent performance across three separate test cohorts [29]. A probability threshold was applied to each pixel to determine its likelihood of being stromal [29]. The resulting probabilistic stromal mask was converted into a binary mask using a specific probability threshold. The AI-based TSP scores (TSP_auto_) were calculated by dividing the number of pixels predicted to be non-tumor (stroma) by the number of pixels predicted to be tumor within the pathologist- marked tumor region on the H&E slides with a probability threshold of 0.5. Using the previously established 50% threshold for the stroma-to-tumor proportion [10–12, 14, 17, 18], TSP_auto_ were converted as high ($\ge$50%) and low (<50%).

### Statistical analysis

Diagnosis, pathology, and treatment information were summarized for all patients using descriptive statistics, including TSP_auto_ and KELIM. Student’s *t* tests or Wilcoxon rank-sum tests were used for continuous variables, while Chi-square or Fisher’s exact tests were applied for categorical variables. Time-to-event data, including OS and PFS, were visualized with Kaplan-Meier survival plots, with survival probabilities and 95% confidence intervals (CIs) estimated for years 1 through 5. Survival distributions by TSP_auto_ and KELIM were compared using log-rank tests. Cox proportional hazard models were used to assess the association of KELIM scores, TSP_auto_, and TSP_manual_ with OS and PFS. Hazard ratios (HRs) and 95% CIs were reported. Adjusted models included stage and resection status. KELIM and TSP scores were summarized based on platinum status and compared using Wilcoxon rank-sum, Chi-square, or Fisher’s exact tests. All reported *p* values are two-sided, with a significance level of 0.05. Statistical analyses were performed using R (version 4.4.1, R Core Team).

### Study approval

This study was approved by the University of Minnesota institutional review board. Patients provided written informed consent for the use of surgical tissue specimens in research. The study was adhered to the Declaration of Helsinki and followed the Transparent Reporting of a Multivariable Prediction Model for Individual Prognosis or Diagnosis guidelines for AI prediction model validation.

## Results

### Patient characteristics

This cohort was collated from 89 patients at the University of Minnesota Masonic Cancer Center with EOC to evaluate the relationship between KELIM and AI-based TSP (see Fig. 1 for general schema). Patient demographics and clinical characteristics including International Federation of Gynecology and Obstetrics (FIGO) stage, platinum resistance status, and residual disease after surgery, are listed in Table 1. Most patients were FIGO stage III (50 patients [56.2%]), and the majority underwent primary surgery followed by adjuvant therapy (53 [59.6%]), and 8 patients (9.2%) had a suboptimal resection (>1 cm of disease) following surgery. The distribution of stage, treatment, and platinum response in this cohort broadly reflects the characteristics of the epithelial ovarian cancer patient population typically seen at diagnosis, consistent with prior reports [21, 30, 31]. Of the 89 patients, 48 (53.9%) had a high TSP_auto_, while 41 had a low TSP_auto_.

**Table 1:** Patient demographics and clinical characteristics of patients divided by AI-based TSP and KELIM.

Characteristics	Patients, No. (%)						
	All (*n* = 89)	TSP < 50% (*n* = 40)	TSP ≥ 50% (*n* = 49)	*P* value^a^	KELIM < 1 (*n* = 54)	KELIM ≥ 1 (*n* = 35)	*P* value^a^
Age at diagnosis (yr), mean (SD)	61.8 (9.5)	62.0 (8.6)	61.6 (10.3)	0.877	61.9 (10.0)	61.5 (8.9)	0.854
KELIM, median (range)	0.9 (0.3, 2.3)	0.9 (0.4, 2.3)	0.8 (0.3, 1.9)	-	0.7 (0.3, 1.0)	1.3 (1.0, 2.3)	-
Histology, n (%)							
High Grade Serous	72 (80.9%)	29 (72.5%)	43 (87.8%)	0.258	41 (75.9%)	31 (88.6%)	0.055
Clear Cell	7 (7.9%)	4 (10.0%)	3 (6.1%)		6 (11.1%)	1 (2.9%)	
Endometrioid	3 (3.4%)	2 (5.0%)	1 (2.0%)		0 (0.0%)	3 (8.6%)	
Mucinous	2 (2.2%)	2 (5.0%)	0 (0.0%)		2 (3.7%)	0 (0.0%)	
Carcinosarcoma	2 (2.2%)	2 (5.0%)	0 (0.0%)		2 (3.7%)	0 (0.0%)	
Mixed	2 (2.2%)	1 (2.5%)	1 (2.0%)		2 (3.7%)	0 (0.0%)	
Undifferentiated	1 (1.1%)	0 (0.0%)	1 (2.0%)		1 (1.9%)	0 (0.0%)	
FIGO Stage, n (%)							
Stage IC	7 (7.9%)	5 (12.5%)	2 (4.1%)	0.177	4 (7.4%)	3 (8.6%)	0.740
Stage IIA	4 (4.5%)	3 (7.5%)	1 (2.0%)		3 (5.6%)	1 (2.9%)	
Stage IIB	5 (5.6%)	3 (7.5%)	2 (4.1%)		4 (7.4%)	1 (2.9%)	
Stage IIIA	3 (3.4%)	2 (5.0%)	1 (2.0%)		3 (5.6%)	0 (0.0%)	
Stage IIIB	15 (16.9%)	8 (20.0%)	7 (14.3%)		10 (18.5%)	5 (14.3%)	
Stage IIIC	32 (36.0%)	9 (22.5%)	23 (46.9%)		18 (33.3%)	14 (40.0%)	
Stage IV	23 (25.8%)	10 (25.0%)	13 (26.5%)		12 (22.2%)	11 (31.4%)	
Timing of Therapy, n (%)							
Adjuvant therapy	53 (59.6%)	26 (65.0%)	27 (55.1%)	0.344	33 (61.1%)	20 (57.1%)	0.709
NACT	36 (40.4%)	14 (35.0%)	22 (44.9%)		21 (38.9%)	15 (42.9%)	
Platinum Status, n (%)							
Sensitive	67 (75.3%)	32 (80.0%)	35 (71.4%)	0.351	37 (68.5%)	30 (85.7%)	0.066
Resistant	22 (24.7%)	8 (20.0%)	14 (28.6%)		17 (31.5%)	5 (14.3%)	
Optimal Resection, n (%)							
Unknown	2	0	2	0.065	2	0	
Yes	79 (90.8)	39 (97.5%)	40 (85.1%)		44 (84.6%)	35 (100.0%)	0.019
No	8 (9.2%)	1 (2.5%)	7 (14.9%)		8 (15.4%)	0 (0.0%)	
Germline *BRCA1/2* and HR status, n (%)							
*BRCA1*-mt	9 (10.8%)	5 (13.2%)	4 (8.9%)	0.755	3 (6.1%)	6 (17.6%)	0.572
*BRCA2*-mt	9 (10.8%)	3 (7.9%)	6 (13.3%)		6 (12.2%)	3 (8.8%)	
*BRCA1/2*-wt, HRD	7 (8.4%)	2 (5.3%)	5 (11.1%)		5 (10.2%)	2 (5.9%)	
*BRCA1/2*-wt, HRP	20 (24.1%)	10 (26.3%)	10 (22.2%)		12 (24.5%)	8 (23.5%)	
*BRCA1/2*-wt, HR unknown	38 (45.8%)	18 (47.4%)	20 (44.4%)		23 (46.9%)	15 (44.1%)	
Unknown	6	2	4		5	1	

*TSP* tumor stroma proportion, *KELIM* k elimination constant of CA-125, *NACT* neoadjuvant chemotherapy, *BRCA* breast cancer gene, *HR* homologous recombination, *mt* mutant, *wt* wild type, *HRD* homologous recombination deficient; homologous recombination proficient.

^a^Comparison of categorical variables were conducted using Chi-square or Fisher’s exact test, and the comparison of age and KELIM score between groups was conducted using Student’s *t*test or Wilcoxon rank-sum test, respectively.

In this cohort, the median KELIM score was 0.9 (range: 0.3–2.3). The majority of patients (60.7%) had an unfavorable KELIM (<1) (Table 1). Patients with an unfavorable KELIM score (<1) were significantly more likely to undergo suboptimal resection following surgery compared to those with a favorable KELIM score (*p* = 0.015) (Table 1). No other significant differences in patient characteristics were observed between the high and low KELIM groups. We further analyzed demographic and clinical characteristics, including mean age at diagnosis, histology, stage, and platinum status, in relation to TSP_auto_ and KELIM score (Table 1).

### KELIM was significantly associated with platinum-resistance status

**Fig. 2:**
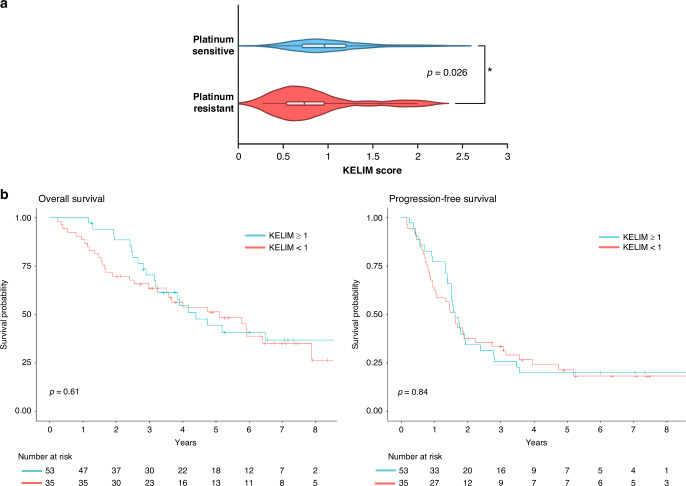
KELIM is significantly associated with platinum status in all patients. **a** Depicts the KELIM score distribution by platinum status—platinum-resistant or sensitive with the median score denoted by center of box-and-whisker plot line. To investigate the association between platinum status and continuous KELIM scores, Wilcoxon rank-sum test was used. For the platinum sensitive group, minimum value was 0.26, lower quartile 0.71, median 0.96, upper quartile 1.2, and maximum value 2.3. The platinum resistant group had minimum value was 0.27, lower quartile 0.52, median 0.74, upper quartile 0.98, and maximum value 2.0. **b** Effect of KELIM on survival outcomes is not significant. Kaplan Meier survival plot for OS and PFS by KELIM. Log-rank test was used. Created in https://BioRender.com.

**Fig. 3:**
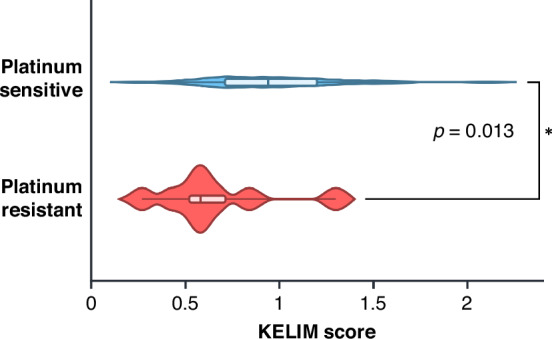
KELIM is significantly associated with platinum status in patients that received adjuvant chemotherapy. KELIM score distribution by platinum status—platinum resistant or sensitive with the median score denoted by line in center of box-and-whisker plot. For the platinum sensitive group, minimum value was 0.26, lower quartile 0.71, median 0.94, upper quartile 1.2, and maximum value 2.1. The platinum resistant group had minimum value was 0.27, lower quartile 0.46, median 0.58, upper quartile 0.80, and maximum value 1.3. To investigate the association between platinum status and continuous KELIM scores, Wilcoxon rank-sum test was used. Created in https://BioRender.com.

We analyzed outcomes by KELIM both as a categorical variable (KELIM≥1 or <1) and as a continuous variable, considering PFS and OS. KELIM was not significantly associated with survival outcomes (Table S1). Survival outcomes remained non-significant in subgroup analyses stratified by treatment timing (adjuvant vs. neoadjuvant therapy, Table S2). KELIM score was also not-significantly associated with survival outcomes even when analyzing only the population with Stage III-IV disease (Table S2). In this cohort, a significant association was observed between KELIM score and platinum status, patients with platinum-resistant tumors were more likely to have an unfavorable KELIM score (p = 0.026, Table 2, Fig. 2). The median KELIM score was 1.0 in patients with platinum-sensitive tumors and 0.7 in platinum-resistant tumors. Among those who underwent upfront surgery followed by adjuvant therapy, KELIM remained significantly associated with platinum status (*p* = 0.013, Fig. 3). Although KELIM was not associated with survival outcomes, patients with an unfavorable KELIM (<1) were significantly more likely to undergo suboptimal resection (*p* = 0.019, Table 1). All patients with suboptimal surgical resection had an unfavorable KELIM, and 75% (6/8 patients) had received neoadjuvant chemotherapy. This suggests that the KELIM score was not solely influenced by CA-125 elevations due to residual disease but may reflect a more aggressive tumor biology or advanced malignancy that limits the feasibility of optimal cytoreductive surgery.

**Table 2:** Continuous KELIM score is associated with platinum status.

Variable	Platinum sensitive (*N* = 67)	Platinum resistant (*N* = 22)	*P* value^a^
KELIM score, median (range)	1.0 (0.3, 2.3)	0.7 (0.3, 2.0)	0.026
KELIM score, *n* (%)			0.066
<1	37 (55.2%)	17 (77.3%)	
≥1	30 (44.8%)	5 (22.7%)	
TSP_manual_, *n* (%)			0.293
<50%	33 (49.3%)	8 (36.4%)	
≥50%	34 (50.7%)	14 (63.6%)	
TSP_auto_, *n* (%)			0.351
<50%	32 (47.8%)	8 (36.4%)	
≥50%	35 (52.2%)	14 (63.6%)	

Comparison of KELIM scores and TSP assessments by platinum status.

^a^To investigate the association between platinum status and continuous KELIM scores, Wilcoxon rank-sum was used. Chi-square tests were used for categorical KELIM scores and TSP_auto/manual_.

### Concordance between AI-based TSP and manual TSP assessments

**Fig. 4:**
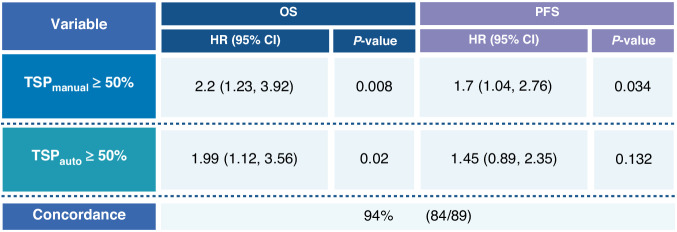
AI-TSP assessment reproduces manual classification of TSP. Summary of Cox proportional hazard models for OS and PFS to investigate the performance of TSP_auto_ compared to TSP_manual_ assessment in their effects on survival outcomes in EOC. Concordance refers to ability of AI to preproduce the classification of pathologist-assessed TSP_._ Hazard ratios and 95% confidence intervals are presented. Created in https://BioRender.com.

To ensure the reliability of AI-based TSP assessments, we compared the case classification of TSP_auto_ to TSP_manual_. TSP_auto_ demonstrated a 94% concordance (84/89 cases) with TSP_manual_ (Cohen’s Kappa 0.89, 95% CI 0.79, 0.98, *p*<0.001). As expected, given this high concordance, TSP_auto_ performed similarly to TSP_manual_. These findings confirm that TSP_auto_ assessments were comparable to TSP_manual_ in their association with OS (Fig. 4).

### AI-based TSP assessment had a significant effect on overall survival in EOC

TSP_auto_ demonstrated a significant association with OS using the log-rank test (*p* = 0.017, Fig. 4). High TSP_auto_ (≥50%) was associated with poorer OS (HR 1.99, 95% CI 1.12–3.56, *p* = 0.02, Fig. 4). The effect of TSP_auto_ on OS remained significant even after adjusting for KELIM score (*p* = 0.036), suggesting its value as an independent prognostic biomarker. After adjusting for stage and resection status, the hazard ratio for OS for high TSP_auto_ was 1.5 (95% CI 0.82–2.75, *p* = 0.192).

**Fig. 5:**
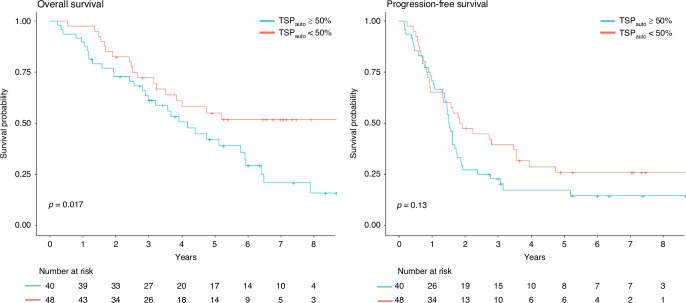
AI-TSP demonstrated a significant effect on overall survival. Kaplan Meier survival plot for OS and PFS by TSP_**auto**_ high or low. Significantly lower overall survival in TSP_auto_ high group (*p* = 0.017), and lower PFS (*p* = 0.13) demonstrated. Log-rank test was used. The corresponding summary table of survival probabilities by year and TSP_auto_ is shown.

**Fig. 6:**
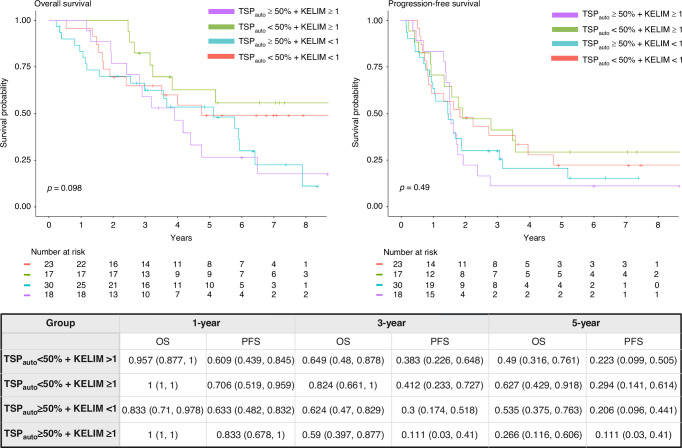
No improved prognostic value in combined categorization of AI-TSP and KELIM on survival outcomes. To explore the combined utility of TSP_auto_ and KELIM, survival outcomes were analyzed based on both stroma-high or low TSP_auto_ assessments and KELIM scores (KELIM ≥ 1 or <1). The Kaplan Meier survival plot for OS and PFS with summary of survival probabilities by year and TSP_auto_ and KELIM are above. There was no significant difference in survival outcomes between curves. Log-rank test was used. Created in https://BioRender.com.

As demonstrated in Figs. 3 and 5, TSP_auto_ had a significant effect on outcomes, whereas KELIM did not. To explore the combined utility of TSP_auto_ and KELIM, we analyzed survival outcomes based on both stroma-high or low TSP_auto_ assessments and KELIM scores (KELIM≥1 or <1) (Fig. 6). Patients with stroma-high TSP_auto_ (≥50%) and unfavorable KELIM score (<1) had the lowest survival probabilities at 2 years, but beyond 3 years the survival curves for high TSP_auto_ and KELIM score <1 cross with the subgroup with high TSP_auto_ and KELIM score (≥1), suggesting the prognostic durability of TSP _auto._ The subgroup with low-stroma TSP_auto_ (<50%) and favorable KELIM score (≥1) had the highest survival probabilities throughout the duration of follow-up (Fig. 6). However, HR for progression and death (Table S1) and survival probabilities (Fig. 6) were not significantly different between the four groups.

## Discussion

Markers for the early treatment response in ovarian cancer can impact clinical and patient decision-making and have the potential to improve patient outcomes. KELIM and TSP are emerging prognostic biomarkers because both have demonstrated early prognostic value for the treatment of EOC [3–5, 7, 9, 17–19], and are cost-effective as they utilize information that already falls within the standard of care for treatment and diagnosis of EOC (i.e. trending CA-125 levels and H&E stained tissue, respectively) [32]. However, the application of TSP has been limited by the need for trained pathologist to perform scoring and the caveat of inter-observer variability in manual pathologist assessments [33, 34]. Additionally, KELIM requires 100 days of therapy to calculate a score. Our study attempts to mediate the limitations of both markers by applying computational pathology to automate TSP scoring and exploring the synergistic effect of TSP at diagnosis with KELIM following treatment.

Our study accomplished several key objectives. Firstly, we established the concordance between manual and AI-based assessments of TSP calculated in EOC. Secondly, we evaluated the association of TSP_manual_ and TSP_auto_ assessments with survival outcomes in EOC patients. Finally, we investigated the association of KELIM in combination with TSP and survival outcomes and platinum sensitivity in EOC.

By evaluating TSP as a biomarker, clinicians can gain valuable insights into the aggressiveness of the tumor, and the potential need for additional targeted or maintenance treatments at the time of tissue diagnosis. With that prognostic guidance, clinicians could theoretically alter therapeutic plans, determine the timing of optimal debulking surgery, offer different maintenance strategies to those at high risk for recurrence or platinum resistance, and better counsel their patients on expectations at the time of initial diagnosis. Thus TSP, if validated following additional rigorous analyses, holds great promise and potential for altering the landscape of EOC treatment from the outset of initial diagnosis. Other widely applied biomarkers in EOC such as BRCA status or homologous recombination deficiency have limitations including expense (costing hundreds of dollars) and longer waiting time for results given the send-out nature of these labs. Additionally, these biomarkers and primarily limited to guiding the use of poly (ADP-ribose) polymerase (PARP) inhibitors as maintenance therapies [35, 36]. Our recent studies [18, 19] and others in the field [16, 20, 37] underscore the pivotal role of TSP in defining the risk profile of patients with EOC, which is also reflected in other cancers. A high stroma content within the TME has been correlated with poorer prognosis and reduced survival rates in a broad range of cancers [16–19, 38–40], which is attributed to dense stroma that can facilitate tumor cell survival, immune evasion, and resistance to therapies by creating a physical and biochemical barrier against therapeutic agents [13, 41, 42]. Thus, incorporating TSP as a biomarker could refine prognostic assessments and guide the development of stroma-targeted therapies, ultimately enhancing personalized treatment strategies for patients with EOC.

Pathologists rely on H&E images to examine tissue samples, identifying cellular structures and abnormalities indicative of cancerous growth [43]. Traditional histopathological evaluation, while effective, is based on qualitative assessments and is subject to interobserver variability and limitations in quantifying complex spatial patterns within the TME [43–46]. Although AI has made significant strides in analyzing H&E images, many AI models function as black boxes and offer limited interpretability [47, 48]. Thus, there is an urgent need to develop explainable biomarkers that not only predict survival outcomes and treatment responses, but explainability also drives large-scale trust and adoption [49].

By evaluating TSP through both manual and AI-based assessments, we have demonstrated a high degree of concordance, thereby affirming the potential of automated approaches in histopathological analysis and reinforcing their clinical utility. Integration of AI-based TSP assessments into clinical practice offers several advantages. It enhances the precision and efficiency of pathologic evaluations, enabling more accurate prognostication and personalized treatment planning. AI-based TSP can reduce time and resource burden on pathology providers and can provide greater reproducibility than manual assessments from varying pathologists. Future research should focus on developing machine learning algorithms capable of performing whole-slide analysis, thereby eliminating the need for manual delineation of the tumor region of interest and reducing dependence on pathologist effort for TSP assessment. In addition, incorporating larger and more diverse datasets will be important to further improve the robustness and generalizability of these methods. Additionally, prospective clinical trials are necessary to validate TSP’s utility as a prognostic biomarker for chemoresistance and to explore its potential in guiding treatment selection.

KELIM has been increasingly studied in EOC, as an attractive biomarker because it is readily available and has minimal cost, and has demonstrated prognostic value during initial treatment [4–7, 9], demonstrated by its recent inclusion in clinical practice guidelines for EOC [50]. Though in our cohort, KELIM was significantly associated with platinum-status, but not with survival outcomes. The bulk of studies on KELIM are in highly selective clinical trial patients [3–6, 9]. Our study suggests that KELIM may not be as generalizable in real-life populations. In addition, TSP provides prognostic value similar to KELIM, but at time of diagnosis rather than following 100 days of initial treatment. In our study KELIM alone was not significantly associated with outcomes but was associated with chemoresistance. These findings suggest a potential association between key outcomes, including survival and platinum resistance, and two biomarkers that are simple, cost-efficient and accessible in early treatment of EOC, highlighting an opportunity to incorporate them into clinical decision-making, though further analyses are needed.

This study has limitations that merit consideration. First, its retrospective design may introduce inherent biases, although we utilized well-established clinical and pathological assessments consistent with standard practice. Second, the cohort size was moderate, which limited the power to detect significant associations for certain biomarkers such as KELIM; however, it was sufficient to confirm our earlier prospective and retrospective findings that manually assessed high TSP is associated with patient outcomes and treatment response [18, 19]. Though the cohort was large enough to detect statistical significance, a larger cohort is needed to more robustly evaluate AI-based TSP’s prognostic value in various histologies that had limited samples (such as clear cell) in this study. Finally, tumor heterogeneity remains a recognized challenge in tissue-based analyses. This study analyzed specimens from primary tumors (ovary/fallopian tube, 49.4%, *N* = 44) when available, or alternatively from metastatic sites including the omentum (34.8%, *n* = 31) or peritoneum (15.7%, *n* = 14). Therefore, the results of our study suggest the TSP can be associated with outcome in a cohort with mixed primary and metastatic sites subjected to analysis. Our study focused on analysis of TSP from a single representative tumor slide as have prior studies on TSP, and we did not attempt to exhaustively compare intra-patient TSP heterogeneity across anatomic locations. Further study is needed to explore the potential TSP variability between various metastatic vs. primary tumor sites within the same patient. In addition, heterogeneity across serial slides within the same tissue block is a potential pitfall, particularly for borderline cases near the 50% threshold. To date, the implications of tumor heterogeneity on TSP assessments has not been fully explored. A pretrained algorithm to assess TSP, such as used in this study, could be utilized to efficiently explore tumor heterogeneity across multiple anatomic sites. Although variability of TSP across tumor sites and post-chemotherapy remains to be further explored, our findings provide important insights into the clinical utility of AI-derived TSP in epithelial ovarian cancer.

In conclusion, our study highlights the importance of TSP in the prognosis of EOC, and even more critically, the high concordance between manual and AI-based TSP assessments validates the reliability of automated approaches in histopathology. The associations of TSP with survival outcomes underscore its potential as a valuable biomarker for personalized treatment strategies, and greater association with outcomes compared to KELIM in this real-world cohort.

## Supplementary information

star_bjc_reports_supplementary

## Data Availability

Supporting data values for all figures can be made availability by request to the corresponding author.
