# Supplementary material for: STAR (stroma-tumor AI risk) assessment: association of AI-derived tumor-stroma proportion with patient survival provides added prognostic value beyond KELIM in epithelial ovarian cancer
Source: BJC Rep. 2026 Feb 6;4:4. doi: 10.1038/s44276-026-00205-1 (PMC12881467; doi:10.1038/s44276-026-00205-1)
Supplement: Supplementary file 1 — star_bjc_reports_supplementary [file 44276_2026_205_MOESM1_ESM.docx]

**Supplementary Information**

**Table S1. Summary of Cox proportional hazard models for OS and PFS for EOC patients**

|  | **OS** | | **PFS** | |
| --- | --- | --- | --- | --- |
| **Variable** | **HR (95% CI)** | **P-value** | **HR (95% CI)** | **P-value** |
| KELIM score ≥1 vs <1 | 0.86 (0.49, 1.52) | 0.611 | 0.95 (0.59, 1.54) | 0.842 |
| Stage III-IV patients only- KELIM score ≥1 vs <1 | 0.92 (0.51, 1.66) | 0.77 | 1.06 (0.64, 1.75) | 0.827 |
| KELIM score  Q33%-Q67% vs min-Q33% | 0.78 (0.41, 1.51) | 0.469 | 0.73 (0.41, 1.3) | 0.28 |
| KELIM score  Q67%-max vs min-Q33% | 0.67 (0.33, 1.33) | 0.252 | 0.82 (0.46, 1.44) | 0.481 |
| KELIM score | 0.66 (0.33, 1.35) | 0.258 | 0.76 (0.4, 1.43) | 0.391 |

Results from individual Cox proportional hazard models to investigate the effect of KELIM score as continuous variable, as categorical (≥1 vs <1 ) and in tertiles (upper, middle and lower third), and TSP_auto_ on OS and PFS for EOC. Hazard ratios and 95% confidence intervals are presented. There was not a significant effect.

|  | **OS** | | **PFS** | |
| --- | --- | --- | --- | --- |
| KELIM score ≥1 vs <1 in: | **HR (95% CI)** | **P-value** | **HR (95% CI)** | **P-value** |
| NACT | 0.53 (0.22, 1.28) | 0.157 | 0.65 (0.31, 1.35) | 0.244 |
| Adjuvant Therapy | 1.1 (0.52, 2.33) | 0.802 | 1.19 (0.62, 2.28) | 0.595 |

**Table S2. Summary of Cox proportional hazard models for OS and PFS of KELIM by treatment type**

Results from individual Cox proportional hazard models to investigate the effect of KELIM score on PFS and OS in patients by either NACT or adjuvant therapy. Hazard ratios and 95% confidence intervals are presented. There was not a significant effect.


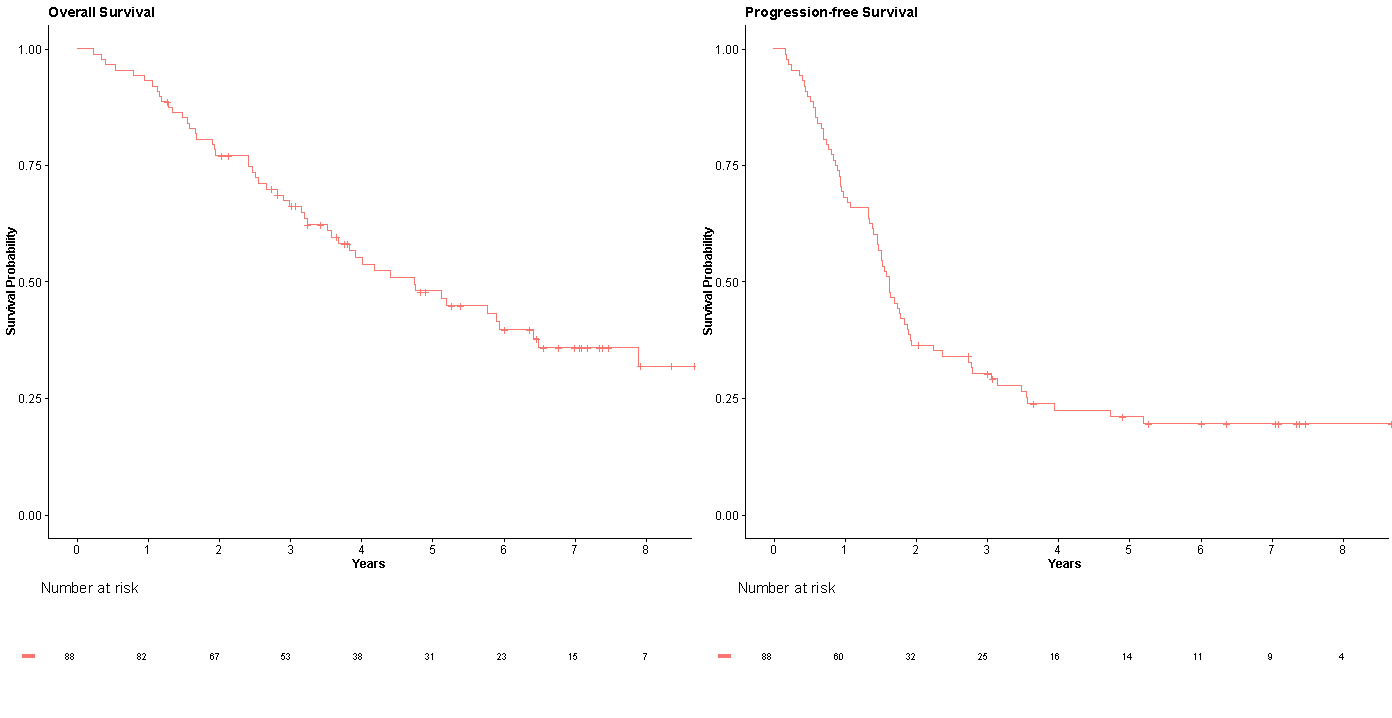


| **Outcome** | **1-year** | **3-year** | **5-year** |
| --- | --- | --- | --- |
| OS | 0.932 (0.881, 0.986) | 0.662 (0.568, 0.77) | 0.48 (0.38, 0.606) |
| PFS | 0.682 (0.591, 0.786) | 0.304 (0.221, 0.418) | 0.21 (0.138, 0.32) |

**Figure S1.** Kaplan Meier plots with summary of survival probabilities of OS ad PFS for all patients.


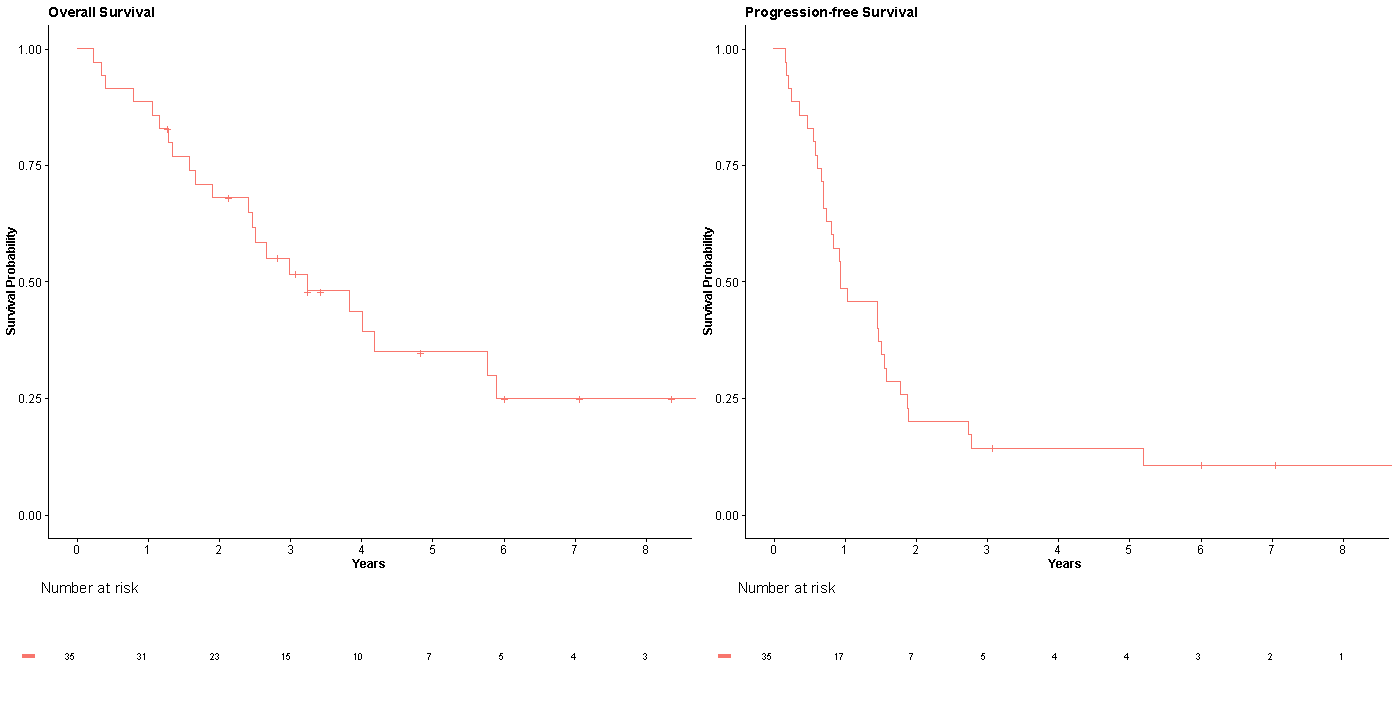


| **Outcome** | **1-year** | **3-year** | **5-year** |
| --- | --- | --- | --- |
| OS | 0.886 (0.786, 0.998) | 0.517 (0.37, 0.721) | 0.349 (0.209, 0.583) |
| PFS | 0.486 (0.345, 0.683) | 0.143 (0.063, 0.322) | 0.143 (0.063, 0.322) |

**Figure S2.** Kaplan Meier plots with summary of survival probabilities of OS and PFS for all patients who received NACT.


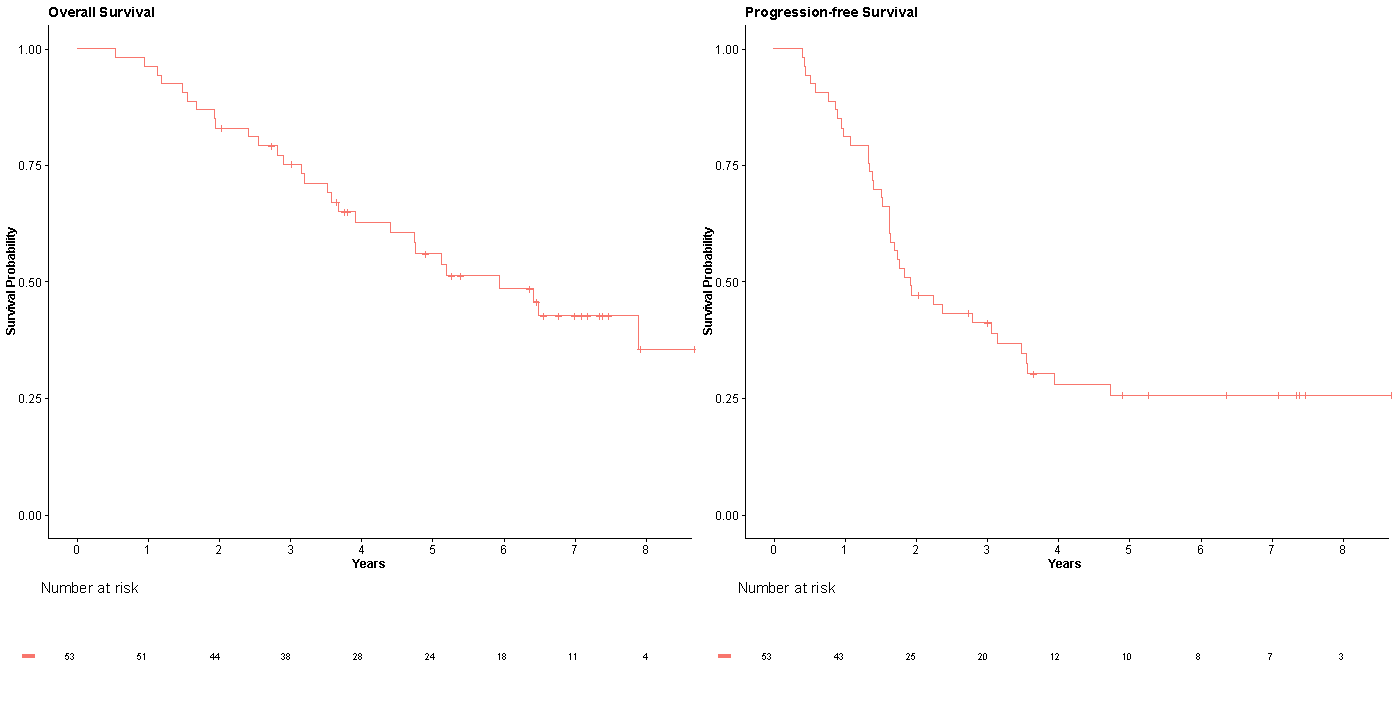


| **Outcome** | **1-year** | **3-year** | **5-year** |
| --- | --- | --- | --- |
| OS | 0.962 (0.912, 1) | 0.752 (0.644, 0.879) | 0.56 (0.437, 0.718) |
| PFS | 0.811 (0.713, 0.924) | 0.412 (0.298, 0.569) | 0.257 (0.159, 0.415) |

**Figure S3.** Kaplan Meier survival plots with summary of survival probabilities of OS and PFS for all patients who received adjuvant chemotherapy.
